# Prevalence and associated factors of chronic constipation among Japanese university students

**DOI:** 10.3389/fpubh.2024.1258020

**Published:** 2024-01-16

**Authors:** Nhu Thi Hanh Vu, Duc Trong Quach, Shunsuke Miyauchi, Mai Ngoc Luu, Mahoko Yoshida, Doan Thi Nha Nguyen, Atsuo Yoshino, Yoshie Miyaka, Yuri Okamoto, Shiro Oka, Toru Hiyama

**Affiliations:** ^1^Department of Internal Medicine, University of Medicine and Pharmacy at Ho Chi Minh City, Ho Chi Minh, Vietnam; ^2^Department of Endoscopy, Hiroshima University Hospital, Hiroshima, Japan; ^3^Health Service Center, Hiroshima University, Higashihiroshima, Japan; ^4^Department of Gastroenterology, Graduate School of Biomedical and Health Sciences, Hiroshima, Japan

**Keywords:** chronic constipation, family history, depression, eating disorders, prevalence, lifestyle factors, sleep duration

## Abstract

**Background:**

Chronic constipation (CC) is one of the most frequently reported gastrointestinal disorders in the general population and a prominent problem among university students. The study aimed to evaluate the prevalence and the associated factors of CC among Japanese university students.

**Methods:**

This cross-sectional study was conducted among university students at Hiroshima University, Japan. Students answered the web questionnaire when making a web reservation for the health checkup (April 1 to May 31, 2023). The web questionnaire consisted of four sections, including baseline characteristics, lifestyle factors, family history of CC, and three scales to assess depression and eating disorders: the Beck Depression Inventory (BDI), Eating Attitudes Test (EAT)-26 and Bulimic Investigatory Test (BITE). CC was diagnosed using Rome IV criteria. The multivariate logistic regression model was used to determine CC-related factors.

**Results:**

Out of 10,500 individuals who participated in the annual health checkup, 7,496 participants answered the web questionnaire, of whom 5,386 answered all the survey questions. The mean age of the students was 21.1 ± 4.1 years. The male-to-female ratio was 1:1.17. The prevalence of CC was 13.7%. Factors significantly associated with CC in the multivariate model were first-degree family members with CC [Odd ratio (OR): 2.77, 95% confidence interval (CI): 2.31–3.31], severe depression according to BDI scale (OR: 2.59, 95% CI: 1.96–3.43), female sex (OR: 2.00, 95% CI: 1.69–2.36), and short sleep duration of 6 hours or less per day (OR: 1.28, 95% CI: 1.09–1.50). Lack of physical exercise tended to be associated with CC (OR: 1.19, 95% CI: 1.00–1.40).

**Conclusions:**

CC is prevalent among Japanese university students. Significant risk factors for CC included the first-degree family history of CC, severe depression, female sex, and short sleep duration. Lack of physical exercise tended to be associated with CC. This may contribute to implementing suitable education health programs, health care professionals, and public health policies to identify individuals at risk for CC to prevent and treat CC effectively.

## Introduction

Chronic constipation (CC) is one of the most commonly reported gastrointestinal disorders in the general population (1). CC significantly negatively affects physical and emotional wellbeing, resulting in substandard health outcomes and diminished quality of life (2–4). Furthermore, individuals suffering from CC have increased medical utilization and financial costs, resulting in an enormous economic and social burden (3, 5, 6).

Meta-analyses showed that CC was widespread across countries, and its prevalence varied among different cross-sectional surveys (7). Based on data from integrative reviews, the reported prevalence of CC in the general adult population ranges from 2 to 27% (8). This wide range is partially attributable to differences in study populations and definitions of constipation used in various epidemiologic studies, despite the internationally accepted Rome criteria for CC diagnosis (9). With a recent upward trend, CC affects ~15 to 23% of females and 11% of males in Asia (10). In Japan, the prevalence of CC is estimated to range between 6.1 and 28.0%, depending on the diagnostic scale used (11, 12).

There are few studies using Rome IV criteria to evaluate the prevalence and risk factors of CC in selected populations. Additionally, in recent years, there have been a growing number of studies evaluating CC on various domestic and international populations but fewer studies on CC and its associated factors among university students. University students represent a significant demographic group affected by CC (13). Long-term constipation can damage students' health in some ways, including facial acne and irritability, hemorrhoids, and other diseases that can interfere with their studies and daily activities (13–15). Understanding the prevalence of CC and its risk factors, particularly among university students, is critical in implementing suitable health interventions, such as prevention programs or activities aimed at reducing the risk factors of CC. Therefore, this study aimed to investigate the prevalence of CC among Japanese university students using the Rome IV criteria and identify its associated factors.

## Materials and methods

### Study design and participants

This cross-sectional study was conducted in accordance with the latest version of the Helsinki declaration. The study was administered to Japanese students at Hiroshima University, a national university located in Higashiroshima and Hiroshima, Japan. There are 12 undergraduate schools and 5 graduate schools in the university. The undergraduate schools include the School of Applied Biological Science, School of Integrated Arts and Sciences, School of Informatics and Data Science, School of Education, School of Economics, School of Engineering, School of Law, School of Letters, School of Science, School of Medicine, School of Dentistry, and School of Pharmaceutical Sciences. The graduate level studies comprise the Graduate School of Humanities and Social Sciences, Graduate School of Advanced Science and Engineering, Graduate School of Integrated Sciences for Life, Graduate School of Biomedical and Health Sciences, and Graduate School of Innovation and Practice for Smart Society.

All the students at Hiroshima University are supposed to take health checkups every year. In the academic year 2023, the total student population was 15,701 individuals. Among them, 10,500 individuals participated in the annual health checkup at the beginning of the academic year (from April 1 to May 31, 2023) and were recruited to participate in this study. Students answered the web questionnaire when making a web reservation for the health checkup. Only participants who completed the survey questionnaire in its entirely were eligible for inclusion in the study. Those who did not provide informed consent were excluded.

### Questionnaire development

The survey questionnaire was initially developed in English and consisted of four sections. The first section collected baseline characteristics of the participants, including age, gender, height, and weight. The second section gathered information on participants' lifestyle factors such as smoking, alcohol consumption, physical exercise, breakfast-eating habits, and average sleep duration. The third section surveyed abdominal symptoms experienced by participants and similar symptoms reported by their first-degree family members. The final section assessed three scales, including the Beck Depression Inventory (BDI), Eating Attitudes Test (EAT)-26, and Bulimic Investigatory Test (BITE) (16–18). The survey questions are of different formats, including yes/no questions, multiple-choice questions (MCQ), and open-ended questions.

A group of healthcare professionals from the Health Service Center of Hiroshima University, including three internists (SM, MY, TH) and three psychologists (AY, YM, YO) carefully revised the original questionnaire. The revised questionnaire was then piloted by 10 medical professionals to ensure its validity. Based on their feedback, the questionnaire was modified and translated into Japanese. A reverse translation process was also performed to ensure the accuracy and consistency between the original and translated versions. A pretest by ten native Japanese speakers was also conducted for the translated version. If required, the survey questionnaire was then modified based on the pretest results. The final English survey questionnaire was detailed in the Supplementary material S1.

### Definition

In this study, participants were clinically diagnosed with CC using Rome IV criteria (19). Specifically, CC was defined as the presence of two or more of the following symptoms persisting for at least 3 months: (1) straining during more than 25% of defecations, (2) lumpy or hard stools during more than 25% of defecations, (3) sensation of incomplete evacuation during more than 25% of defecations, (4) sensation of anorectal obstruction or blockage during more than 25% of defecations, and (5) fewer than three spontaneous bowel movements per week.

Obesity was defined as having a body mass index (BMI) ≥ 25 kg/m^2^. Non- alcohol consumption was identified as either never drinking or drinking alcohol once per month or less. The absence of physical exercise was reported if individuals did not engage in exercise for a minimum duration of 20 min per day. Breakfast skipping was described if the participant did not consume breakfast at all during the week. All these lifestyle factors were recorded within the last 6 months.

The BDI scale is a well-known 21-item self-rating tool used to evaluate the main symptoms of depression. A score of 30 or greater on the BDI is considered indicative of severe-to-extreme depression (16).

The EAT-26 is a psychological self-assessing tool used to identify the presence of eating disorders that require professional attention. A score of 20 or above on the EAT-26 indicates the presence of a probable eating disorder (17).

The BITE is a 33-item self-rating scale to assess binge-eating and purging behavior. A total score of 25 and above on the BITE suggests a possible case of binge-eating (18).

### Ethical considerations

The informed consent was embedded in the first page of the online survey questionnaire and was collected from all participants before data collection. Ethical approval for this study was obtained from the Ethical Committee of Hiroshima University, Japan (ethical numbered E-143-3). The collected survey questionnaires were completely anonymous and not revealed to any external entity.

### Statistical analysis

The collected data were organized in an Excel spreadsheet (Microsoft et al., USA) and analyzed using SPSS software version 20.0 (SPSS Inc., Chicago, IL). In the descriptive statistics section, we compare the difference between the CC and non-CC groups using the student's *t*-test and chi-square test. A multivariable logistic regression analysis was applied to investigate the associated factors of CC among participants. A *p*-value of < 0.05 was considered statistically significant.

## Results

### Characteristics of participants

**Figure 1:**
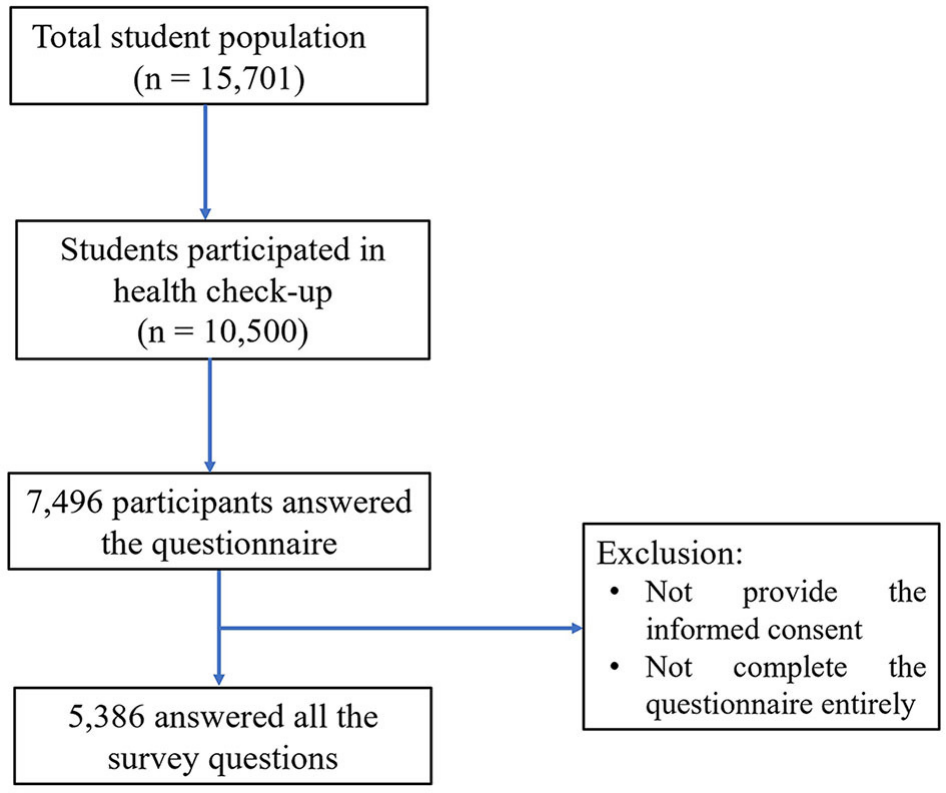
Flow diagram of student recruitment.

Of 10,500 individuals who participated in the annual health checkup, 7,496 participants answered the web questionnaire (Figure 1). Among them, 5,386 answered all the survey questions. According to the Rome IV criteria, the prevalence of CC was 13.7%.

**Figure 2:**
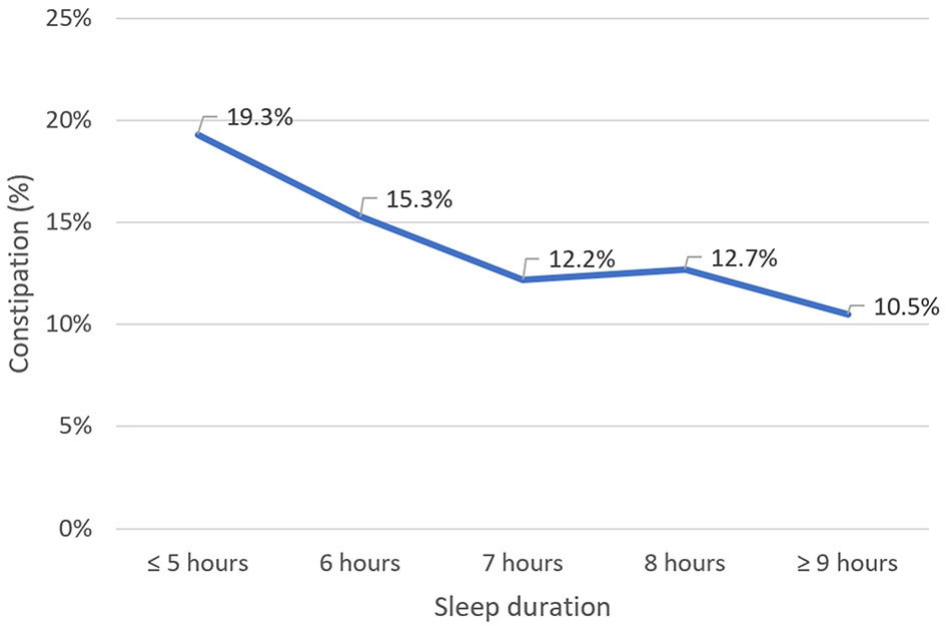
Sleep duration of participants. CC, Chronic constipation.

Table 1 summarizes the characteristics of participants, divided into CC and non-CC groups. The mean age of the participants was 21.1 ± 4.1 years. The male-to-female ratio was 1:1.17. Regarding lifestyle habits, the rate of alcohol consumption and smoking among students was 1.2 and 4.4%, respectively. In addition, 70.1% of students reported engaging in exercise at least 1 day a week. Further, the majority of students slept between 6 and 8 h a day, with a higher proportion of students with CC sleeping <6 h than other students (Figure 2). Regarding breakfast habits, 29.9% of students did not have the habit of eating breakfast. In terms of the BDI scale, 88.1% of students had no depression or mild depression, 9.6% had moderate depression, and 2.3% had severe depression.

**Table 1:** Characteristics of participants.

**Characteristics**	**Total (%) *N* = 5386**	**Participants without CC (%)*N* = 4648**	**Participants with CC (%) *N* =738**	***p*-value**
**Sex**				
Male	2,904 (53.9)	2,636 (56.7)	268 (36.3)	<0.001
Female	2,482 (46.1)	2,012 (43.3)	470 (63.7)	
**Obesity**				
No	4,945 (91.8)	4,263 (91.7)	682 (92.4)	0.53
Yes	440 (8.2)	384 (8.3)	56 (7.6)	
**Alcohol consumption**				
No	5,323 (98.8)	4,593 (98.8)	730 (98.9)	0.82
Yes	63 (1.2)	55 (1.2)	8 (1.1)	
**Smoking**				
No	5,150 (95.6)	4,438 (95.5)	712 (96.5)	0.22
Yes/ever	236 (4.4)	210 (4.5)	26 (3.5)	
**Family history of CC**				
No	4,518 (83.9)	4,019 (86.5)	499 (67.6)	<0.001
Yes	868 (16.1)	629 (13.5)	239 (32.4)	
**Physical exercises**				
No	1,613 (29.9)	1,346 (29.9)	267 (36.2)	<0.001
Yes	3,773 (70.1)	3,302 (71)	471 (63.8)	
**Breakfast**				
No	692 (12.8)	606 (13)	86 (11.7)	0.23
Yes	4,694 (87.2)	4,042 (87)	652 (88.3)	
**Sleep duration**				
≤ 6 h/day	2,326 (43.2)	1,963 (42.2)	363 (49.2)	<0.001
>6 h/day	3,060 (56.8)	2,685 (57.8)	375 (50.8)	
**BDI**				
0–18	5,087 (94.4)	4,437 (95.5)	650 (88.1)	
19–29	238 (4.4)	167 (3.6)	71 (9.6)	<0.001
≥30	61 (1.1)	44 (0.9)	17 (2.3)	0.001
**EAT-26**				
<20	5,330 (99)	4,605 (99.1)	725 (98.2)	0.037
≥20	56 (1)	43 (0.9)	13 (1.8)	
**BITE**				
<20	5,325 (98.9)	4,607 (99.1)	718 (97.3)	<0.001
≥20	61 (1.1)	41 (0.9)	20 (2.7)	

CC, chronic constipation; BDI, Beck Depression Inventory; EAT-26, Eating Attitudes Test-26; BITE, Bulimic Investigatory Test.

### Factors associated with CC

Univariate analysis showed that the prevalence of CC was significantly higher in females than in males (18.9 vs. 9.2%, *p* < 0.001). There was also a significant increase in the rate of CC among participants who had first-degree family members of CC (27.5%), compared to the non-CC group (11%) with *p* < 0.001. The participants with no physical exercise had a higher rate of CC than those who were physically more active (16.6 vs. 12.5%, *p* < 0.001). Sleep duration of 6 h or less was also a risk factor for CC compared with students who slept more than 6 h (15.6 vs. 12.3%, *p* < 0.001). Additionally, the prevalences of depressive and eating disorders according to BDI, EAT-26, and BITE scales were significantly higher in the CC group than in the non-CC group (*p* = 0.001, *p* = 0.037, and *p* < 0.001, respectively).

Table 2 displays the results of the multivariate logistic regression models. The factors significantly associated with CC in the multivariate model were first-degree family members with CC (OR: 2.77, 95% CI: 2.31–3.31), severe depression according to BDI scale (OR: 2.59, 95% CI: 1.96–3.43), female gender (OR: 2.00 (95% CI: 1.69–2.36), and short sleep duration of 6 h or less per day (OR: 1.28, 95% CI: 1.09–1.50). Lack of physical exercise tended to be associated with CC (OR: 1.19, 95% CI: 1.00–1.40).

**Table 2:** Multivariable logistic regression analysis of factors associated with CC.

**Risk factors**	**OR**	**95% CI**	***p*-value**
Family history of CC	2.77	2.31–3.31	<0.001
BDI ≥ 30	2.59	1.96–3.43	<0.001
Female sex	2.00	1.69–2.36	<0.001
Short sleep duration (≤ 6 h per day)	1.28	1.09–1.5	0.003
No physical exercises	1.19	1–1.4	0.05
BITE ≥ 20	1.63	0.88–3.04	0.12
EAT-26 ≥ 20	1.08	0.53–2.23	0.83

CC, chronic constipation; OR, odd ratio; CI, confidence interval; BDI, Beck Depression Inventory; EAT-26, Eating Attitudes Test; BITE, Bulimic Investigatory Test.

In our study, there were no significant differences in the prevalence of CC among university students regarding obesity, drinking alcohol, smoking, and breakfast consumption (*p* > 0.05).

## Discussion

To the best of our knowledge, this is the first and largest questionnaire-based study investigating the prevalence and associated factors of CC among Japanese university students. Our survey revealed that 13.7% of Japanese university students met the Rome IV criteria for diagnosis of CC. This finding is most consistent with those reported previously in the general population. Suares et al. conducted a systematic review and meta-analysis containing 261,040 subjects and determined a global prevalence of CC of 14 % (1). Based on Rome criteria, another recent systematic review and meta-analysis found that pooled prevalence of CC was 15.3% using Rome I, 11.2% using Rome II, 11.4% using Rome III, and 10.1% using Rome IV (7). Previous studies on college and university students in China and Tunisia reported that the prevalence of CC ranged from 5.1 to 11.4%, which was lower than our finding (20–22). However, a higher prevalence (23.9%) of CC was found in the Australian adult population (23). Variations in CC prevalence estimates across studies can be attributed to differences in data collection, constipation definitions, and sampling methods.

CC is a disorder with multiple causes, including intestinal motility dysfunction, visceral sensitivity, anorectal musculature, and enteric nervous system (24). Numerous factors have been identified as potential contributors to constipation based on previous studies. It is widely accepted that prime risk factors for constipation in the community include age, sex, education level, physical activity, lifestyle, and psychological factors (1, 25–27).

It was established that a history of abdominal pain or bowel problems in first-degree relatives is highly related to irritable bowel syndrome; however, there is limited information regarding CC (28). In this study, participants with first-degree family history of CC had an increased risk of CC. In contrast to our results, Lock et al. conducted a self-report questionnaire and reported that first-degree relatives with abdominal pain or bowel problems were significantly associated with irritable bowel syndrome and dyspepsia but not constipation, diarrhea, or gastroesophageal reflux (29). Similarly, a population-based cohort by Chang et al. also demonstrated no evidence of familial aggregation in adults from the community with CC (30). However, one study based on an interview questionnaire on same-sex twins in Australia suggested a substantial genetic component of functional bowel disorders and that the results seemed unlikely to be explained by bias (31). Moreover, a similar diet and lifestyle may also contribute to the increased risk of CC in the family. Further studies on their relationship with CC are needed.

Our study observed a relationship between depression and CC. Individuals with severe depression based on the BDI assessment had a 2.59-fold greater risk of CC than the non-CC group. This result is consistent with a previous review of comorbidities in CC patients indicating that depression was the most commonly reported psychiatric disorder associated with CC, occurring in 15–29% of CC patients (32–34). Although the underlying mechanism for psychological problems has not been fully understood, it is suggested that these disorders can alter gut-brain interaction and induce changes in gastrointestinal motility (35). Additionally, Rajindrajith et al. observed that children or adolescents with CC had more emotional and behavioral disturbances (36, 37). University students are in a transitional period between campus life and social life, as well as an essential stage of physical and mental growth. Some stressful life events, such as significant changes in their life schedules, poor physical condition, some frustrating events, and other events, such as boredom with school, loss of love, and arguments or fights with others (38), may lead to gastrointestinal dysfunction, and increase the likelihood of CC. In addition, university students with CC may also experience negative emotions such as anxiety as a result of CC. Consequently, paying attention to university students' life stress and spiritual and psychological health is one of the essential measures for preventing and treating CC.

Regarding the prevalence of CC between sex, our study demonstrated that females had a higher prevalence than males. The current finding is consistent with previous studies indicating a higher prevalence of CC among females (1, 25, 39). This increased prevalence in females could be attributed to hormonal fluctuations, dietary habits, and a history of physical or emotional problems (40, 41). Elevated levels of progesterone hormone have been observed to induce a reduction in the motility and muscle tone of the gastrointestinal tract, leading to constipation (42). In addition, males have a greater skeletal muscle mass and a longer anal sphincter than females. The increased anterolateral abdominal wall musculature in males may allow for increased abdominal pressure during defecation. Male individuals also exhibit elevated sphincter resting pressure and squeeze pressure in comparison to their female counterparts (43).

Sleep plays a crucial role in sustaining the body's physiological functions and good health. In our study, the prevalence of CC increased as nighttime sleep duration decreased. Logistic regression analysis showed that university students were more likely to develop CC when their nighttime sleep duration was <6 h. A multicenter study in China demonstrated that older adults who slept for <6 h were at a higher risk of developing constipation (44). This is also consistent with some prior research on younger participants (45–47). Similarly, several studies indicated that poor sleep increased the risk of constipation among college students (48). Furthermore, a study investigating functional gastrointestinal disorders and sleep duration revealed that constipation was related only to reduced sleep rather than other sleep disorders (49).

The connections between bowel movements and physical activity have been a topic of researches for decades. Some studies have demonstrated that exercise stimulates colonic motility and accelerates gastrointestinal transit (50, 51). In our study, lack of physical exercise tended to be associated with CC in university students. Iovino et al. also indicated that physical inactivity was a possible cause of CC in healthy individuals (52). Moreover, according to the American Gastroenterological Association's 2013 technical review on constipation, physical inactivity is a risk factor for constipation, and mild exercise increases intestinal gas clearance (53). However, Wilson concluded that self-reported physical inactivity is not significantly associated with CC (54). Therefore, this would be a crucial research concern for future studies of our group.

Our study has several limitations. First, the participants were recruited exclusively from one national university in Japan, thus limiting the generalizability of findings. Second, the information was obtained through an internet-based self-administered survey; hence, the potential for recall bias was inevitable. Thirdly, due to the cross-sectional design, a causal relationship between CC and risk factors could not be determined. Fourth, other factors such as dietary habits, comorbid conditions, and medication histories were not considered in this study. Moreover, the available data about undergraduate and graduate students, as well as science and art students in our study were insufficient and constrained; therefore, we could not analyze the relationship between prevalence of constipation and these factors. Fifth, the survey response rate in our study is <50%. Students who experienced constipation were probably more inclined to respond. Therefore, further population-based studies with larger sample sizes, long-term follow-up, and better questionnaire designs are required to identify cause-effect relationships between CC and these factors and potential interventions for CC.

## Conclusion

In conclusion, we found that the prevalence of CC among Japanese university students was 13.7%. First-degree family history of CC, severe depression (BDI score ≥ 30), female sex, and short sleep duration (<6 h/day) were significantly associated with CC in this population. Lack of physical exercise tended to be associated with CC. These findings may be of assistance in raising awareness about the significance and risk factors of CC among Japanese university students. This may contribute to implementing suitable education health programs, health care professionals, and public health policies to identify individuals at risk for CC to prevent and treat CC more effectively.

## Data availability statement

The raw data supporting the conclusions of this article will be made available by the authors, without undue reservation.

## Author contributions

NV: Data curation, Formal analysis, Writing—original draft. DQ: Formal analysis, Supervision, Writing—review & editing. SM: Data curation, Writing—original draft. ML: Formal analysis, Writing—original draft. MY: Data curation, Writing—original draft. DN: Formal analysis, Writing—original draft. AY: Data curation, Formal analysis, Writing—review & editing. YM: Data curation, Formal analysis, Writing—review & editing. YO: Data curation, Writing—review & editing. SO: Supervision, Writing—review & editing. TH: Conceptualization, Formal analysis, Supervision, Writing—review & editing.
