# Supplementary material for: Prevalence and associated factors of chronic constipation among Japanese university students
Source: Front Public Health. 2024 Jan 16;12:1258020. doi: 10.3389/fpubh.2024.1258020 (PMC10824902; doi:10.3389/fpubh.2024.1258020)
Supplement: Supplementary file 1 [file Table_1.DOCX]

**QUESTIONNAIRE FOR ASSESSMENT ON CHRONIC CONSTIPATION IN JAPANESE UNIVERSITY STUDENTS**

Please cooperate with the questionnaire survey

The information obtained will not be used for any purpose other than the research.

Please put a check (✓) in the box.

**Age:** ……… years old **Sex:** □Male 　□Female

**Height:** ……………cm **Weight:** ………………kg

**Questions about your lifesyle during the last 6 months**

**Q1:** Do you smoke?

□Yes

□ No, but I used to

□ Never

**Q2**: Do you drink alcohol?

□ ≥ 4 times/week

□ < 4 times/week

□ Never or ≤ 1 time/month

**Q3**: Do you do exercise?

□ ≥ 4 days/week

□ < 4 days/week

□ No

**Q4**: Do you eat breakfast?

□ ≥ 4 days/week

□ < 4 days/week

□ No

**Q5:** How many hours do you sleep on average?

□ Less than 5 hours

□ About 6 hours

□ About 7 hours

□ About 8 hours

□ More than 9 hours

**Questions about your abdominal symtoms**

**Q6**: Have you had two or more of the following ① to ⑤ in the last 3 months? ①Straining during more than ¼ (25%) of defecations ②Lumpy or hard stools more than ¼ (25%) of defecations ③Sensation of incomplete evacuation more than ¼ (25%) of defecations ④Sensation of anorectal obstruction/blockage more than ¼ (25%) of defecations ⑤Fewer than three spontaneous bowel movements per week

□ Yes

□ No

**Q7:** Does anyone in your family (your parents, your brothers, your sisters) have the same symtoms in the last 3 months?

□ Yes

□ No

**Q8: Beck Depression Inventory (BDI)**

On this questionnaire are groups of statements. Please read all the statements in a given group. Then pick out at least one statement in each group which describes you best in terms of this past week. Be sure to read all the statements in each group before making your choice(s).

**Q8.1:**

0 I do not feel sad.

1 I feel sad.

2 I am sad all the time and I can't snap out of it.

3 I am so sad and unhappy that I can't stand it.

**Q8.2:**

0 I am not particularly discouraged about the future.
1 I feel discouraged about the future.
2 I feel I have nothing to look forward to.
3 I feel the future is hopeless and that things cannot improve.

**Q8.3:**

0 I do not feel like a failure.
1 I feel I have failed more than the average person.
2 As I look back on my life, all I can see is a lot of failures.
3 I feel I am a complete failure as a person.

**Q8.4:**

0 I get as much satisfaction out of things as I used to.
1 I don't enjoy things the way I used to.
2 I don't get real satisfaction out of anything anymore.
3 I am dissatisfied or bored with everything.

**Q8.5:**

0 I don't feel particularly guilty
1 I feel guilty a good part of the time.
2 I feel quite guilty most of the time.
3 I feel guilty all of the time.

**Q8.6:**

0 I don't feel I am being punished.
1 I feel I may be punished.
2 I expect to be punished.
3 I feel I am being punished.

**Q8.7:**

0 I don't feel disappointed in myself.
1 I am disappointed in myself.
2 I am disgusted with myself.
3 I hate myself.

**Q8.8:**

0 I don't feel I am any worse than anybody else.
1 I am critical of myself for my weaknesses or mistakes.
2 I blame myself all the time for my faults.
3 I blame myself for everything bad that happens.

**Q8.9:**

0 I don't have any thoughts of killing myself.
1 I have thoughts of killing myself, but I would not carry them out.
2 I would like to kill myself.
3 I would kill myself if I had the chance.

**Q8.10:**

0 I don't cry any more than usual.
1 I cry more now than I used to.
2 I cry all the time now.
3 I used to be able to cry, but now I can't cry even though I want to.

**Q8.11:**

0 I am no more irritated by things than I ever was.
1 I am slightly more irritated now than usual.
2 I am quite annoyed or irritated a good deal of the time.
3 I feel irritated all the time.

**Q8.12:**

0 I have not lost interest in other people.
1 I am less interested in other people than I used to be.
2 I have lost most of my interest in other people.
3 I have lost all of my interest in other people.

**Q8.13:**

0 I make decisions about as well as I ever could.
1 I put off making decisions more than I used to.
2 I have greater difficulty in making decisions more than I used to.
3 I can't make decisions at all anymore.

**Q8.14:**

0 I don't feel that I look any worse than I used to.
1 I am worried that I am looking old or unattractive.
2 I feel there are permanent changes in my appearance that make me look unattractive
3 I believe that I look ugly.

**Q8.15:**

0 I can work about as well as before.
1 It takes an extra effort to get started at doing something.
2 I have to push myself very hard to do anything.
3 I can't do any work at all.

**Q8.16:**

0 I can sleep as well as usual.
1 I don't sleep as well as I used to.
2 I wake up 1-2 hours earlier than usual and find it hard to get back to sleep.
3 I wake up several hours earlier than I used to and cannot get back to sleep.

**Q8.17:**

0 I don't get more tired than usual.
1 I get tired more easily than I used to.
2 I get tired from doing almost anything.
3 I am too tired to do anything.

**Q8.18:**

0 My appetite is no worse than usual.
1 My appetite is not as good as it used to be.
2 My appetite is much worse now.
3 I have no appetite at all anymore.

**Q8.19:**

0 I haven't lost much weight, if any, lately.
1 I have lost more than five pounds.
2 I have lost more than ten pounds.
3 I have lost more than fifteen pounds.

**Q8.20:**

0 I am no more worried about my health than usual.
1 I am worried about physical problems like aches, pains, upset stomach, or constipation.
2 I am very worried about physical problems and it's hard to think of much else.
3 I am so worried about my physical problems that I cannot think of anything else.

**Q8.21:**

0 I have not noticed any recent change in my interest in sex.
1 I am less interested in sex than I used to be.
2 I have almost no interest in sex.
3 I have lost interest in sex completely.

**Q9.** **Eating Attitudes Test (EAT-26)**

This is a screening measure to help you determine whether you might have an eating disorder that needs professional attention. This screening measure is not designed to make a diagnosis of an eating disorder or take the place of a professional consultation. Please fill out the below form as accurately, honestly and completely as possible. There are no right or wrong answers.

| **Q9.1. Check a response for each of the following statements:** | | | Always | Usually | Often | Some  times | Rarely | Never |
| --- | --- | --- | --- | --- | --- | --- | --- | --- |
| 1. | | Am terrified about being overweight. | □ | □ | □ | □ | □ | □ |
| 2. | | Avoid eating when I am hungry. | □ | □ | □ | □ | □ | □ |
| 3. | | Find myself preoccupied with food. | □ | □ | □ | □ | □ | □ |
| 4. | | Have gone on eating binges where I feel that I may not be able to stop. | □ | □ | □ | □ | □ | □ |
| 5. | | Cut my food into small pieces. | □ | □ | □ | □ | □ | □ |
| 6. | | Aware of the calorie content of foods that I eat. | □ | □ | □ | □ | □ | □ |
| 7. | | Particularly avoid food with a high carbohydrate content (i.e. bread, rice, potatoes, etc.) | □ | □ | □ | □ | □ | □ |
| 8. | | Feel that others would prefer if I ate more. | □ | □ | □ | □ | □ | □ |
| 9. | | Vomit after I have eaten. | □ | □ | □ | □ | □ | □ |
| 10. | | Feel extremely guilty after eating. | □ | □ | □ | □ | □ | □ |
| 11. | | Am preoccupied with a desire to be thinner. | □ | □ | □ | □ | □ | □ |
| 12. | | Think about burning up calories when I exercise. | □ | □ | □ | □ | □ | □ |
| 13. | | Other people think that I am too thin. | □ | □ | □ | □ | □ | □ |
| 14. | | Am preoccupied with the thought of having fat on my body. | □ | □ | □ | □ | □ | □ |
| 15. | | Take longer than others to eat my meals. | □ | □ | □ | □ | □ | □ |
| 16. | | Avoid foods with sugar in them. | □ | □ | □ | □ | □ | □ |
| 17. | | Eat diet foods. | □ | □ | □ | □ | □ | □ |
| 18. | | Feel that food controls my life. | □ | □ | □ | □ | □ | □ |
| 19. | | Display self-control around food. | □ | □ | □ | □ | □ | □ |
| 20. | | Feel that others pressure me to eat. | □ | □ | □ | □ | □ | □ |
| 21. | | Give too much time and thought to food. | □ | □ | □ | □ | □ | □ |
| 22. | | Feel uncomfortable after eating sweets. | □ | □ | □ | □ | □ | □ |
| 23. | | Engage in dieting behavior. | □ | □ | □ | □ | □ | □ |
| 24. | | Like my stomach to be empty. | □ | □ | □ | □ | □ | □ |
| 25. | | Have the impulse to vomit after meals. | □ | □ | □ | □ | □ | □ |
| 26. | | Enjoy trying new rich foods. | □ | □ | □ | □ | □ | □ |
| **Q9.2. Behavioral Questions:**  **In the past 6 months have you:** | | | Never | Once a month or less | 2-3 times a month | Once a week | 2-6 times a week | Once a day or more |
| A | Gone on eating binges where you feel that you may not be able to stop? * | | □ | □ | □ | □ | □ | □ |
| B | Ever made yourself sick (vomited) to control your weight or shape? | | □ | □ | □ | □ | □ | □ |
| C | Ever used laxatives, diet pills or diuretics (water pills) to control your weight or shape? | | □ | □ | □ | □ | □ | □ |
| D | Exercised more than 60 minutes a day to lose or to control your weight? | | □ | □ | □ | □ | □ | □ |
| E | Lost 20 pounds or more in the past 6 months | | Yes □ | | No □ | |  | |
| ***** Defined as eating much more than most people would under the same circumstances and feeling that eating is out of control | | | | | | | | |

**Q10. Bulimic Investigatory** **Test (BITE)**

**Q10.1.** Do you have a regular daily eating pattern?

Yes=0, No=1

**Q10.2.** Are you a strict dieter?

Yes=0, No=1

**Q10.3.** Do you feel a failure if you break your diet once?

Yes=0, No=1

**Q10.4.** Do you count the calories of everything you eat, even when not on a diet?

Yes=0, No=1

**Q10.5.** Do you ever fast for a whole day?

Yes=0, No=1

***Q10.6**. . . . If yes, how often is this?

EVERY SECOND DAY 5

2 – 3 TIMES A WEEK 4

ONCE A WEEK 3

NOW AND THEN 2

HAVE ONCE 1

***Q10.7**. Do you do any of the following to help you lose weight? (click the number)

|  | Never | Occasionally | Once a week | 2 -3 times a week | daily | 2 – 3 times a day | 5+ times a day |
| --- | --- | --- | --- | --- | --- | --- | --- |
| TAKE DIET PILLS | 0 | 2 | 3 | 4 | 5 | 6 | 7 |
| TAKE DIURETICS | 0 | 2 | 3 | 4 | 5 | 6 | 7 |
| TAKE LAXATIVES | 0 | 2 | 3 | 4 | 5 | 6 | 7 |
| MAKE YOURSELF VOMIT | 0 | 2 | 3 | 4 | 5 | 6 | 7 |

**Q10.8.** Does your pattern of eating severely disrupt your life?

Yes=0, No=1

**Q10.9.** Would you say that food dominated your life?

Yes=0, No=1

**Q10.10.** Do you ever eat and eat until you are stopped by physical discomfort?

Yes=0, No=1

**Q10.11.** Are there times when all you can think about is food?

Yes=0, No=1

**Q10.12.** Do you eat sensibly in front of others and make up in private?

Yes=0, No=1

**Q10.13.** Can you always stop eating when you want to?

Yes=0, No=1

**Q10.14.** Do you ever experience ***overpowering*** urges to eat and eat and eat?

Yes=0, No=1

**Q10.15.** When you are feeling anxious do you tend to eat a lot?

Yes=0, No=1

**Q10.16.** Does the thought of becoming fat ***terrify*** you?

Yes=0, No=1

**Q10.17.** Do you ever eat large amounts of food rapidly (not a meal)?

Yes=0, No=1

**Q10.18.** Are you ashamed of your eating habits?

Yes=0, No=1

**Q10.19.** Do you worry that you have no control over how much you eat?

Yes=0, No=1

**Q10.20.** Do you turn to food for comfort?

Yes=0, No=1

**Q10.21.** Are you able to leave food on the plate at the end of a meal?

Yes=0, No=1

**Q10.22.** Do you deceive other people about how much you eat?

Yes=0, No=1

**Q10.23**. Does how hungry you feel determine how much you eat?

Yes=0, No=1

**Q10.24.** Do you ever binge on large amounts of food?

Yes=0, No=1

**Q10.25**. . . . If yes, do such binges leave you feeling miserable?

Yes=0, No=1

**Q10.26.** If you do binge, is this only when you are alone?

Yes=0, No=1

**Q10.27**. If you do binge, how often is this?

HARDLY EVER 1

ONCE A MONTH 2

ONCE A WEEK 3

2 – 3 TIMES A WEEK 4

DAILY 5

2 – 3 TIMES A DAY 6

**Q10.28**. Would you go to great lengths to satisfy an urge to binge?

Yes=0, No=1

**Q10.29**. If you overeat do you feel ***very*** guilty?

Yes=0, No=1

**Q10.30.** Do you ever eat in secret?

Yes=0, No=1

**Q10.31.** Are your eating habits what you would consider to be normal?

Yes=0, No=1

**Q10.32.** Would you consider yourself to be a compulsive eater?

Yes=0, No=1

**Q10.33.** Does your weight fluctuate by more than 5 pounds in a week?

Yes=0, No=1
